# The Security Rating on Local Ablation and Interventional Therapy for Hepatocellular Carcinoma (HCC) and the Comparison among Multiple Anesthesia Methods

**DOI:** 10.1155/2019/2965173

**Published:** 2019-02-19

**Authors:** Qianhui Jin, Xinhua Chen, Shusen Zheng

**Affiliations:** ^1^Division of Hepatobiliary Pancreatic Surgery, Department of Surgery, The First Affiliated Hospital, College of Medicine, Zhejiang University, Zhejiang Province, Hangzhou, China; ^2^Key Laboratory of Combined Multi-Organ Transplantation, Ministry of Public Health, Key Laboratory of the Diagnosis and Treatment of Organ Transplantation, CAMS Key Laboratory of Organ Transplantation, Zhejiang Province, Hangzhou, China; ^3^Collaborative Innovation Center for Diagnosis Treatment of Infectious Diseases, Zhejiang Province, Hangzhou, China

## Abstract

Recently, the interventional therapies are used more often in clinical practice for hepatocellular carcinoma. The most commonly used methodologies include radiofrequency ablation, microwave ablation, laser ablation, and cryotherapy. Most of the interventional operations need local anesthesia combined with intravenous sedation. Also, some interventional therapy centers apply general anesthesia. However, different anesthesia methods can cause diverse effects on patients' pain management, recovery time, and hospitalization time. For the better understanding of the current anesthesia application status, we summarize and analyze multiple anesthesia methods while being applied in interventional therapy for hepatocellular carcinoma; in addition, their characters are also compared in this paper.

## 1. Introduction

As a curative therapy, the interventional ablations are used more often in clinical practice for hepatocellular carcinoma, e.g., radiofrequency ablation, microwave ablation, laser ablation, and cryotherapy. Most of the interventional operations need local anesthesia combined with intravenous sedation or general anesthesia. For better understanding of the current anesthesia application status and diverse effect, the multiple anesthesia methods applied in interventional therapy for hepatocellular carcinoma are reviewed.

## 2. The Development of Interventional Therapy for Hepatic Carcinoma

Hepatocellular carcinoma (HCC) is a malignant tumor with high global incidence which is ranked as the 7th highest incidence among malignant tumors in male and 5th in female [1]. In China, primary hepatocellular carcinoma has the 2nd highest incidence in malignant tumors and the mortality is in the third position among global malignant tumors [2]. At present, the main radical therapies for hepatocellular carcinoma include surgical resection and liver transplantation; however, most of the hepatocellular carcinoma patients have had cirrhosis, lymphatic metastasis, or distant metastasis when they were diagnosed; therefore, 70% of those patients have already lost the opportunity of getting radical surgery. With regard to those hepatocellular carcinomas which are impossible to be radically resected, such as advanced hepatocellular carcinoma and small hepatocellular carcinoma, interventional therapy has been noticed as an increasingly significant adjuvant method of treating advanced HCC and a radical therapy for small HCC. It is featured by the small trauma and obvious curative effect, providing patients who are intolerant to surgical resection a chance to be radically cured. If 2-3 neoplastic foci spread deep in the liver or are central type (less or equal to 5 centimeters), the effect of local ablation is similar to surgical resection which can achieve radical ablation after mini-invasive treatment. The local ablation therapy for hepatocellular carcinoma is applicable for treating patients whose tumor is smaller than 5 cm; whose tumor nodes are less than 3 and the diameter of the largest tumor is smaller than 3 cm; and whose tumors have no vessels, bile ducts, and adjacent organ invasion and metastasis and liver function is Child-Pugh A or B. As for those patients having single or multiple tumors with a diameter of 3 cm to 7 cm, further TACE should be considered.

### 2.1. Radiofrequency Ablation (RFA)

Radiofrequency ablation can ablate tumor by heating local liver tissues causing tumors' necrosis. For small hepatocellular carcinoma, especially with a diameter smaller than 5 centimeters, RFA has an equal effect to surgical resection, which is recognized as one of the efficient approaches to radically cure hepatocellular carcinoma [3]. Hocquelet et al. [4] compared the efficacy of monopole radiofrequency ablation and nontouch bipolar radiofrequency ablations, finding that bipolar radiofrequency ablation can destruct local tumor. Kawamura et al. [5] studied the nontouch bipolar radiofrequency ablative effect on the relapse of single tumor and small hepatocellular carcinoma and found that it reduces the tumor recurrence compared with touch radiofrequency ablations. The curative effect and complication of radiofrequency ablations depend on tumors' anatomy positions. When hepatocellular carcinoma is close to vessels, bile ducts, liver capsule, and other adjacent organs, the ablation-caused complications, such as great vascular injury [6], rise.

### 2.2. Microwave Ablation (MWA)

Microwave ablation transfers high-frequency electromagnetic energy to microwave radiation energy, and then, the heat causes coagulative necrosis and eliminates tumors. Xiang et al. [7] and Hui-xiong and Ming-yan [8] compared the effects of RFA and MWA on treating hepatocellular carcinoma. They concluded that there is no obvious difference between MWA and RFA in terms of curative effect and complications. Livraghi [9] found there is no difference between MWA's complications and RFA's; they both have a low rate of serious complications [10] and no death cases; thus, they are safe therapies to treat hepatocellular carcinoma.

### 2.3. Cryotherapy

Cryotherapy is a therapy to kill tumor with low temperature. After technical upgrade, its curative effect has been significantly improved, and its complications have been substantially decreased. In addition, percutaneous minimally invasive approach has had replaced the quondam open surgery. Orlacchio et al. [11] retrospectively studied the feasibility and safety of percutaneous cryoablation guided by B-mode ultrasound and CT, and they found 4 patients' tumors were eliminated efficiently without complications which meant cryotherapy is potent, safe, and feasible. Dunne et al. [12] found there is no statistical difference between cryotherapy and RFA by comparing complications. Both of them are safe.

### 2.4. Transcatheter Arterial Chemoembolization (TACE)

The major chemical ablation method to interventional therapy for hepatocellular carcinoma is TACE. It infuses chemotherapeutic drugs and embolic agents through the hepatic artery to deprive the blood supply of the tumor. This therapy is the first fine choice of chemical ablation [13]. TACE is suitable for high vascular hepatocellular carcinoma. It is able to reduce tumors' size to meet the surgical indication so that radical surgical therapy can be operated. It can also be used in patients with multiple or locally unresectable hepatocellular carcinoma, or postoperative recurrence and postoperative recurrence prevention. Xue et al. [14] found that TACE causes less-severe complications after being applied to massive hepatocellular carcinoma, which proved that it is a safe and efficient therapy. Besides, patients with better preoperative hepatic function and lower alpha fetoprotein level can achieve better curative results and prolonged survival.

## 3. Curative Evaluation and Follow-Up

The local therapy is evaluated by CT, MRI, and B-mode ultrasound. The effect can be classified as complete ablation and incomplete ablation. If patients have incomplete ablation, they can be operated again. If after twice ablation, still, patients have tumors, the ablation therapy should be considered failed; it should be abandoned and replaced by other therapies.

When the tumors are completely ablated, patients should get a periodic follow-up. Usually, every 2-3 months, the tumor marker test, color Doppler ultrasound, MRI, or CT should be done so as to discover potential recrudescent local tumor focuses and new focuses within the life in time.

## 4. Comparison among Multiple Anesthesia Methods in Interventional Therapy for Hepatocellular Carcinoma

### 4.1. Local Anesthesia

Local anesthesia has a certain antiproliferative effect on the tumor. Because simple local anesthesia cannot satisfy the analgesic requirement, more interventional therapies for hepatocellular carcinoma are performed under local anesthesia combined with intravenous analgesia. However, pain controls is often difficult to manage, especially in some cases where the tumor is large or in a special position, for example, under the diaphragm or near the liver capsule [15, 16]. If intraoperative pain is not well controlled, interventional operation will be affected. On the other hand, local anesthesia is simple and convenient, with fewer postoperative complications, and there is no need for resuscitation.

### 4.2. General Anesthesia

Although interventional therapy for hepatocellular carcinoma is local treatment, the requirement for anesthesia is slightly higher. Several situations, for example, the pain control during the intervention, the ache by tumor necrosis after operation, and the respiratory activity's impact on the diaphragm activity, would also affect the accuracy of operators, which could influence the effect of treatment and the time of operation. General anesthesia has a good analgesic effect. It can reduce the intraoperative pain caused by body movement which may influence the treatment effect. Besides, during the general anesthesia, using appropriate anesthetics can reduce the dosage of intraoperative drugs, shorten the time to resuscitation, and improve patient recovery.

Currently, local anesthesia is still widely used in interventional therapy; sometimes intravenous analgesics are used to relieve intraoperative pain. While using lidocaine local anesthesia, the intraoperative blood pressure and heart rate are increased, affecting patients' hemodynamic stability. And intraoperative pain is often difficult to control. The position of the tumor also influences pain scoring [16]. For example, the tumor located at the septum or under the capsule is an important factor; the tumor diameter [17] is also an important factor affecting blood pressure and heart rate of patients with large tumors changing more than those with small tumors during the treatment. Moreover, local anesthesia may affect respiratory activity and cause anesthesia-related complications, such as respiratory depression or respiratory arrest. Applying intravenous general anesthesia can ensure more stable hemodynamics, blood pressure, and heart rate compared with using local anesthesia. General anesthesia's incidence of postoperative complications such as pleural effusion and gas accumulation is lower than that of the local anesthesia group as well. Compared with local anesthesia, intravenous general anesthesia has a longer postoperative recovery time, but intraoperative hemodynamics is more stable. And local anesthesia [18] has less postoperative complications and shorter hospitalization time. The comparison between local anesthesia and general anesthesia is shown in Table 1. And the comparison of complications is shown in Table 2.

## 5. The Effect of Different Anesthetics on Interventional Therapy for Hepatocellular Carcinoma

With the application of abundant new anesthetics, more literature has compared the effects of different anesthetics in interventional therapy.

### 5.1. The Effect of Sevoflurane on Interventional Therapy for Hepatocellular Carcinoma

Sevoflurane is a type of inhalation anesthetic which has the advantages of fast induction, muscle relaxation, and rapid postoperative recovery. In the past researches, for hepatocellular carcinoma surgery or interventional therapy, sevoflurane has little influence on hepatic function and is relatively safe. Nishiyama et al. [19] found that sevoflurane and isoflurane have a certain effect on hepatic function after hepatectomy, but sevoflurane's is less. Yi et al. [20] studied the anesthetic safety of sevoflurane inhalation anesthesia and intravenous anesthesia with propofol in microwave ablation. Propofol is a routine surgical anesthetic. The adverse drug effects of that are mainly on the inhibition of the respiratory system and the cardiovascular system; therefore, increasing the dose of propofol will reduce the security of anesthesia. Compared with propofol and sevoflurane inhalation anesthesia, cardiovascular and respiratory depression is not obvious; hemodynamics and respiration are more stable, and induction of anesthesia is more smooth. However, the incidence of nausea and vomiting after sevoflurane is higher than that of propofol which could affect the electrolyte balance of the body and cause food to be inhaled into the trachea. Ling-xi et al. [21] did research on the postoperative effect of different densities of sevoflurane on hepatectomy. It turned out that the usage of sevoflurane in surgical anesthesia could reduce the dosage of propofol, accelerate the recovery and extubation after the operation, and reduce the postoperative complications. Song et al. [22] compared sevoflurane and propofol's effects on hepatic function finding that both of them have no obvious difference on the peak value of hepatic function items such as aminotransferase, bilirubin, and alkaline phosphatase, which indicates that, in clinical application, they have similar effects on hepatic function.

### 5.2. The Effect of Dexmedetomidine on Interventional Therapy for Hepatocellular Carcinoma

Dexmedetomidine is a kind of alpha-2 receptor agonist and highly selective agonist which has analgesic, sedative, and antianxiety functionality, and it can maintain hemodynamic stability, widely used in general anesthesia. A large amount of research indicated that dexmedetomidine, to some degree, can protect organs, such as myocardial cell. Sulaiman et al.'s research [23] showed that dexmedetomidine can reduce intraoperative blood pressure and heart rate. It can prevent cardiac ischemia by regulating the blood flow of the heart, which has a certain protective effect on the heart. Wang et al.'s [24] study found that dexmedetomidine did not affect immune function, stabilize hemodynamics, and reduce inflammatory response. Feld et al.'s study [25] has shown that dexmedetomidine can replace fentanyl's analgesic effect and reduce blood pressure and heart rate. Some studies [26–28] show the effect of dexmedetomidine combined with that of sufentanil during perioperation and found that the use of dexmedetomidine can reduce the dosage of anesthetics and the sedation level is higher. During the operation, the group in which patients use dexmedetomidine has lower blood pressure and heart rate. It may be related to the increment of vagus tension after using dexmedetomidine. Nonetheless, the incidence of postoperative complications like respiratory depression, nausea, and vomiting is lower than that of those without using medetomidine. Meanwhile, it does not increase adverse reactions such as hypertension and tachycardia. It effectively reduces the proportion of postoperative complications. And it can increase the security of anesthesia. Joung et al. [29] found that compared with propofol, medetomidine can maintain respiratory stability and reduce the dosage of anesthetics in radiofrequency ablation.

### 5.3. The Effect of Opioids on Interventional Therapy

Opioids are good analgesic drugs, widely used in clinical anesthesia. They can inhibit cellular immunity and humoral immunity, affecting the recurrence rate of tumors after the surgery [30]. Studies [31, 32] show that using fentanyl and sufentanil during perioperation will not affect postoperative recurrence. Studies [33] show that compared with general anesthesia combined with epidural anesthesia, there is no significant difference in the survival rate and recurrence rate of general anesthesia combined with morphine intravenous analgesia. Yi et al. [34] studied the application of remifentanil combined with propofol intravenous anesthesia in radiofrequency ablation for hepatocellular carcinoma, and they found that remifentanil could reduce the recovery time, decrease the dosage of propofol in anesthesia, and lower the fluctuation of mean arterial pressure. But it will increase the risk of apnea and add respiratory management during the surgery. Guohua and Li [35] found that patients maintained their awareness during the treatment, which made them possible to follow doctors' instructions and the operation would not be disturbed by the patient's unconscious movements. Besides, it not easy to get synergies with other drugs, and thus, the effects of sedation and analgesia not meeting the requirements of surgery will not happen. Li-hong et al. [36] studied the application of propofol combined with remifentanil in radiofrequency ablation for hepatocellular carcinoma. It is found that the combination of them is helpful to maintain the stability of blood pressure and heart rate during the treatment, and the degree of sedation in the operation makes the patient slightly uncomfortable. The patient can follow the doctor's instructions in the treatment which helps the treatment to proceed smoothly. Lingyan et al. [37] compared the effects of tramadol and fentanyl in the treatment of hepatocellular carcinoma by microwave ablation. They found that if patients use fentanyl combined with propofol during the operation, the proportion of hypotension, bradycardia, and hypoxemia was higher. Tramadol affects blood circulation less than fentanyl does, which means it is relatively secure.

## 6. The Effect of Anesthesia on Therapy Result

### 6.1. The Effect of Anesthesia on Immune Function

Various methods of anesthesia and anesthetic drugs have effects on immune function after operation. Using anesthetic methods and drugs with less influence on postoperative immune function has certain advantages for patients' curative effect and tumor's metastasis and recurrence after the surgery. In surgery for hepatocellular carcinoma, Sun et al. [38] found that compared with intravenous inhalation-combined anesthesia, after the surgery for hepatocellular carcinoma, there is a significant increase in the amount of CD4+/CD8+ T cells when patients use general anesthesia combined with epidural anesthesia. The two different anesthesia ways have obvious difference effects. The study found that general anesthesia combined with epidural anesthesia can reduce immunosuppression and speed up the recovery of immune function after the surgery. Fu et al.'s [39] study found that patients with combined general anesthesia and epidural anesthesia have more stable immune function than those with intravenous anesthesia. Wada [40] found that sevoflurane could reduce the metastasis of cancer cells and reduce postoperative immunosuppression. Moreover, compared with general anesthesia with sevoflurane, sevoflurane general anesthesia combined with spinal block anesthesia can reduce immunosuppression and the metastasis of cancer cells. Huimin et al. [41] found that propofol anesthesia can influence the level of serum interleukins and HSP70 when patients were treated with radiofrequency ablation for hepatocellular carcinoma. And they found that the increased level of IL-6, IL-8, and HSP70 in patients who had got propofol anesthesia was lower than that in the local anesthetic group, which could reduce the nociceptive inflammation of patients. However, in Cho et al.'s study [42], propofol-remifentanil anesthesia can preserve NKCC to achieve good immunity compared with sevoflurane-remifentanil anesthesia. Levins et al. [43] compared immune cell markers for general anesthesia opioid analgesia with propofol-epidural anesthesia and found no significant difference between the two.

### 6.2. The Effect of Anesthesia on Coagulation Function

We can judge hepatic blood coagulation function by comparing general anesthesia's and epidural anesthesia's effect on blood coagulation function; through comparing coagulation indexes (prothrombin time, partial thromboplastin time, platelet count, and more), 3 days before and after the operation, we can compare the variation of the coagulation function. Using the hemostasis test and fibrinolysis test (vWF, tPA, sP-selectin, PAI-1, D-dimer, and PF1z2), we compare the effects of those two methods of anesthesia before and after the operation. Compared with general anesthesia, after 3 days of the operation, those indexes mentioned above restored and got close to the normal range when patients use epidural anesthesia. It was also found that epidural anesthesia can make coagulation indexes of patients with hepatocellular carcinoma restore quicker and can maintain hemostatic stability better.

### 6.3. Pain Management

Local ablation and interventional procedures for hepatocellular carcinoma should follow the WHO three-step analgesic therapy principle and NCCN adult cancer pain guideline. 80% to 90% cancer patients' pain symptoms can be alleviated by standard and effective therapies.

## 7. The Key Points for Safe Operations of the Ablation for Hepatocellular Carcinoma

Local ablation and interventional operations for hepatocellular carcinoma should follow the guidelines for the therapy of hepatocellular carcinoma, and operators should get strict training and sufficient accumulation of practice. Before the treatment, the patient's overall condition and liver function should be assessed comprehensively and adequately. Besides, we should assessed tumor size, location, and number location of tumors and their adjacent organs, formulating reasonable puncture path and ablation scope, and achieve enough safety edge. Based on the size and position of tumors, choosing appropriate imaging-guided techniques (ultrasound or CT) and ablation methods (RFA, MWA, or PEI) is emphasized. In addition, the distance from tumors to the common bile duct and the left and right hepatic ducts should be at least 5 mm. It is not recommended to treat tumor foci that are larger than 5 cm only by ablation. For multiple tumor foci or larger tumors, according to the patient's hepatic function, we can apply TACE combined with ablation treatment before the therapy and it is superior to simple ablation therapy. In order to get the “safe edge” and kill the tumors thoroughly, the ablation range should include 5 mm paracarcinoma tissues. And for invasive carcinoma or metastatic neoplastic foci whose boundary is clear and the shape is irregular, if the condition of adjacent liver tissues and structure is suitable, it is suggested that the ablation range should be expended properly.

## Figures and Tables

**Table 1:** Comparison between local anesthesia and general anesthesia.

	Advantage	Disadvantage
Local anesthesia	No need for recovery [44]	No satisfy the analgesic requirement [15, 16]
	Simple, convenient [44]	More postoperative complications [45]
	Shorter hospitalization time [18]	

General anesthesia	Satisfy the analgesic requirement [45]	Longer postoperative recovery time [18, 45]
	Stable heart rate and blood pressure	

**Table 2:** Comparison of local anesthesia and general anesthesia complications in interventional therapy.

	Local anesthesia	General anesthesia
Complications	Unstable blood pressure [45]	Hypotension [46, 47]
	Unstable heart rate [45]	Slow heart rate [47]
	Pleural effusion [15, 45]	Respiratory depression
	Poor pain control [17]	Postoperative nausea and vomiting [47, 48]
